# Factors associated with the presence of anxiety and depression symptoms in rural hypertensive adults in Bangladesh: leveraging extreme gradient booster machine learning algorithm

**DOI:** 10.3389/fpsyg.2025.1650667

**Published:** 2025-09-10

**Authors:** Zarin Raihana, Manzur Kader, Md. Zahidul Islam, Farzana Akhter Bornee, Md. Nazrul Islam Mondal, Mohammad Rocky Khan Chowdhury, Baki Billah

**Affiliations:** ^1^Department of Clinical Psychology, Faculty of Biological Sciences, University of Rajshahi, Rajshahi, Bangladesh; ^2^Department of Medical Science, School of Health and Welfare, Dalarna University, Falun, Sweden; ^3^Department of Public Health, First Capital University of Bangladesh, Khulna, Bangladesh; ^4^Institute of Biological Sciences, University of Rajshahi, Rajshahi, Bangladesh; ^5^Department of Pediatrics, Bangabandhu Sheikh Mujib Medical University, Dhaka, Bangladesh; ^6^Department of Population Science and Human Resource Development, University of Rajshahi, Rajshahi, Bangladesh; ^7^Department of Epidemiology and Preventive Medicine, School of Public Health and Preventive Medicine, Monash University, Melbourne, VIC, Australia

**Keywords:** anxiety, depression, hypertension, machine learning, logistic regression

## Abstract

**Introduction:**

Anxiety and depression are common among hypertensive patients and can lead to significant health complications. This study aimed to use Extreme Gradient Boosting (XGB) machine learning (ML) technique to select associated factors of anxiety and depression symptoms among people with hypertension in rural areas.

**Methodology:**

A cross-sectional study was conducted using a multistage cluster random sampling. The anxiety and depression symptoms were evaluated using the Generalized Anxiety Disorder-7 (GAD-7) and Patient Health Questionnaire-9 (PHQ-9) scales, respectively. A chi-square test was performed to assess prevalence. XGB model was employed to predict the presence of anxiety and depression symptoms using 13 variables, and the model’s performance was compared with that of the traditional logistic regression (LR) model. Influential variables were explained and ranked using SHapley Additive exPlanations (SHAP) technique.

**Results:**

Among the 496 rural hypertensive adults, approximately 5.9% and 6.4% experienced the presence of anxiety and depression symptoms, respectively. Anxiety and depression symptoms were more prevalent among higher educated patients (14.0%) and who used tobacco (12.4%), respectively. The XGB model demonstrated improved predictive performance (for anxiety, ROC for XGB: 93.1%; for depression, ROC for XGB: 90.7%) compared to the LR model (for anxiety, ROC for LR: 83.8%; for depression, ROC for XGB: 79.7%) in predicting both outcomes. Marital status, body mass index (BMI), cardiovascular disease (CVD), educational status, family history of hypertension and employment were the influential factors in predicting the presence of anxiety symptoms. Similarly, chewing tobacco, family history of hypertension, marital status, CVD, sex, and educational status are important factors in predicting the presence of anxiety.

**Conclusion:**

In Bangladesh, around 6% rural individuals with hypertension experienced the presence of anxiety and depression symptoms. Educational status, marital status, CVD and family history of hypertension were key factors linked to both outcomes. Future research is needed to validate these findings.

## Introduction

In the area of global mental health, anxiety and depression disorders are recognized as prevalent challenges of considerable concern (Kalin, 2020). According to the World Health Organization, in 2019, around 26.6% of the world’s population faced anxiety, and 28.6% had depression (WHO, 2022; Tasneem et al., 2023). In the same year, depressive and anxiety disorders both ranked among the top 25 leading causes of burden worldwide (Vos et al., 2020; Collaborators, 2022). Mental disorders, including anxiety and depression, play a substantial role in this statistic, accounting for approximately 14.3% of worldwide deaths, or roughly 8 million lives each year (Walker et al., 2015). In South Asia, the point prevalence of anxiety and depression are 12% and 16% respectively, and Bangladesh reflects this regional trend (Vidyasagaran et al., 2023). In 2019, approximately 18.7% of Bangladeshi adults face mental health disorders, with 6.7% was experiencing depression and 4.7% dealing with anxiety (NIMH, 2019). Anxiety and depression frequently give rise to the onset of several noncommunicable diseases such as hypertension, diabetes, and cardiovascular diseases (CVDs) (Meng et al., 2012; Rubio-Guerra et al., 2013; Zhang et al., 2025). Further, the coexistence of depression and anxiety with hypertension creates additional challenges, leading to severe CVDs or mortality (Kwapong et al., 2023). Conversely, hypertension causes chronic inflamation which can negatively affect mental health (Kretchy et al., 2014). The prevalence of anxiety and depression among individuals with hypertension in Bangladesh remains largely unexplored. In contrast, a study from China reported a 25.7% prevalence of depressive disorders among patients with essential hypertension (Ruan et al., 2022).

Establishing a causal relationship between anxiety, depression, and hypertension can be challenging. However, overlooking the factors associated with anxiety and depression among people with hypertension may hinder efforts to address the burden of hypertension. In Bangladesh, where over 25% of the population is affected by hypertension, the health burden is particularly severe in rural areas, which are home to 68.5% of the population (Khan et al., 2021). These communities face an increasing prevalence of hypertension, significantly impacts both health and livelihoods. This issue is further compounded by limited access to healthcare, inadequate media coverage, and a lack of information resources (Masuda Akter, 2023). Moreover, the absence of mental health care services highlights the urgent need for comprehensive mental health support in these areas (Hasan et al., 2021).

Despite these challenges, the prevalence and factors associated with symptoms of anxiety and depression among individuals with hypertension in rural Bangladeshi areas remain largely unexplored and poorly understood. However, in some developing countries, such as Morocco, Nepal, Afghanistan, and Ethiopia, hospital-based data were used to assess the prevalence and factors of anxiety and depression among people with hypertension (Abdisa et al., 2022; Hamrah et al., 2018; Shah et al., 2022; Boukhari et al., 2024). Additionally, in Bangladesh, the prevalence, socio-demographic factors, and clinical factors of anxiety and depression have been extensively studied in specific subgroups, such as students, female adolescents, and pregnant women, while largely neglecting individuals with hypertension (Rabby et al., 2023; Islam et al., 2020; Nishat et al., 2023; Nasreen et al., 2011).

Regardless of any specific cohort, such as individuals with hypertension, sociodemographic and clinical factors impact anxiety and depression through complex and interconnected pathways. Younger or older age groups may experience heightened vulnerability due to life-stage stressors or reduced resilience, while gender differences, particularly higher prevalence among females, are linked to biological and social role disparities (Carmel, 2019; Martin et al., 2001; Kirkbride et al., 2024). Lower education, unemployment, and low income contribute to chronic stress, limited access to resources, and reduced coping capacity (Kirkbride et al., 2024; Yang et al., 2024; Santiago et al., 2011). Marital status also plays a role, as lack of social support from being single, divorced, or widowed can increase loneliness and emotional distress (Vaingankar et al., 2020; Zhang et al., 2024). Health-related conditions such as chronic illness, disability, and substance use further exacerbate psychological burden by increasing dependency, physical limitations, and maladaptive coping mechanisms (Desalegn et al., 2023; Parkinson et al., 2023; Magee and Connell, 2021). These factors operate through pathways like prolonged psychosocial stress, social isolation, and reduced access to care, ultimately disrupting mood regulation and cognitive function, and increasing the risk of anxiety and depression.

Anxiety and depression are mostly explained using theoretically informed variables and further most of these studies predominantly relied on traditional regression models in assessing these variables (Rabby et al., 2023; Islam et al., 2020; Nishat et al., 2023; Nasreen et al., 2011). Machine learning (ML) models benefit from both data-driven and theoretically informed variable selection. ML-based selection identifies complex, nonlinear patterns and interactions that may not be apparent through traditional approaches (Steele et al., 2018; Dehghan and Khosravian, 2024; Jabbari and Bigdeli, 2023), especially in high-dimensional datasets. However, incorporating theoretically informed variables ensures that the model includes clinically or contextually relevant factors, enhancing interpretability and credibility. When combined, theory can guide the initial selection by narrowing the focus to meaningful predictors, while ML techniques refine this set by detecting patterns specific to the data. This integrated approach leads to more accurate, robust, and generalizable models that are both data-responsive and grounded in domain knowledge.

Moreover, the use of ML to predict symptoms of anxiety and depression among individuals with hypertension in Bangladesh is limited and has placed less emphasis on identifying associated factors (Moon et al., 2021; Ahmed et al., 2020; Nayan et al., 2022; Rois et al., 2021). Therefore, this study aims to address the existing gap by identifying the factors associated with the presence of anxiety and depression symptoms among people with hypertension in rural Bangladesh using an advanced ML technique namely XGB algorithms. Previous studies have demonstrated the strong predictive performance of the XGB model in predicting mental health conditions (Mardini et al., 2025; Qi and Huang, 2025). The XGB algorithm is a powerful ML framework recognized for its efficiency, flexibility, and portability (Chen and Guestrin, 2016). It operates as an ensemble learning method built on the gradient boosting framework, where models are constructed sequentially to enhance the performance of previous iterations by minimizing errors through the gradient descent algorithm (Chen and Guestrin, 2016). XGB was chosen due to its relatively straightforward interpretability and its ability to perform feature selection during model development. It offers several advantages, making it a strong alternative to traditional statistical methods and other ML algorithms (Qi and Huang, 2025).

## Methods

### Study design and sampling

A cross-sectional study was conducted from August to November 2021, using multistage cluster random sampling to recruit participants from 18 villages in a randomly selected district in Bangladesh.

**Figure 1:**
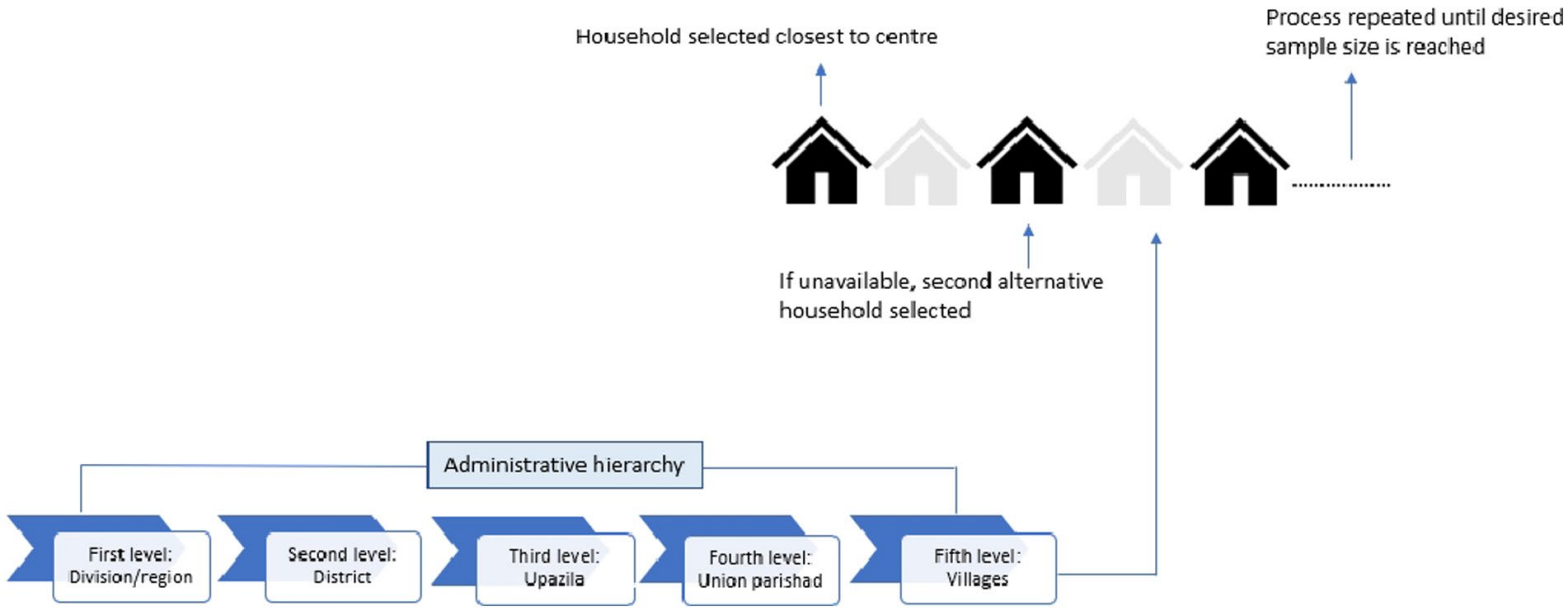
Schematic diagram of sample section.

Bangladesh is divided into eight administrative regions, or divisions: Barisal, Chittagong, Dhaka, Khulna, Mymensingh, Rajshahi, Rangpur, and Sylhet. The study began with the random selection of Khulna division, followed by Jhenaidah district (one of 10 districts in Khulna), and then Jhenaidah Sadar Upazila (one of six Upazilas in the district). Finally, Naldanga Union (one of 14 Unions in Jhenaidah Sadar) was randomly chosen. Participants were recruited from 18 villages within Naldanga Union, excluding four villages after reaching the target sample size (Figure 1).

This study followed ‘Kish Grid’ method to select a household and study participants (Kish, 1949). The ‘Kish Grid’ method was used to select one individual per household for interviews (Supplementary Text S1). The first household was chosen based on proximity to the Union Parishad centre. Following predefined criteria (adults aged 18 or older residing in the selected household and provided written consent), one member was interviewed. Pregnant women, diagnosed mentally impaired individuals, and those who had surgery within the past 3 months were excluded. If the selected member declined or the household was inaccessible, it was marked as a refusal, and the next household was approached. Data collection aimed for proportional representation by ensuring balanced participation across sexes (male and female) and age groups (young adults, adults, middle-aged, and older adults).

### Survey management

The recruitment process of field personnel and training workshop: Five interviewers with social science backgrounds and prior data collection experience, along with two local support persons, were recruited. A registered nurse was trained to measure anthropometrics and blood pressure. Details were presented in Supplementary Text S2.

Data collection instruments: Data was collected through face-to-face interviews using a validated semi-structured questionnaire. The original English questionnaire was translated into Bengali to ensure local comprehension and facilitate data collection (Supplementary Text S3).

Pilot survey: A pilot study was conducted with 24 participants from the selected sampling area to assess the acceptability and feasibility of the questionnaire (Supplementary Text S4).

Quality assurance: To ensure the quality of data collection, several measures were implemented: (i) pre-testing the questionnaire before the survey, (ii) organizing a pre-survey workshop, (iii) monitoring the data collection process, and (iv) using durable equipment Supplementary Text S5). Details of safety measures and data access and storage are provided in Supplementary Text S6, S7.

### Sample size calculation/power analysis

The required formula for determining the sample size: *n* = *Z*^2^_1 − *α*/2_ × *p* (1-*p*) ÷ *d*^2^. Here, *n* represents the required sample size, *p* is the prevalence of hypertension in rural Bangladesh (approximately 27% based on a recent study), and *d* is the desired accuracy level (set at 3%) (Khan et al., 2021). With a standard normal deviate value (*Z*_1 − *α*/2_) of 1.96 for a 95% confidence level, the initial sample size was calculated as 841 participants. To ensure nationwide generalizability and account for the population’s socio-demographic diversity, the sample size was adjusted using a design effect of 1.54 to address sampling variance from the multi-stage study design. (Henry, 1990; Islam et al., 2016; Siddiquea et al., 2023). The final sample size was determined as 1,472. A minimum of 80 participants were interviewed in each village. However, information was collected from 1,603 participants in the actual study to adjust non-response rate. Among them, 496 participants were found to have hypertension.

### Blood pressure measurement and define hypertension

Blood pressure (BP) was measured after participants abstained from coffee and smoking for at least 30 min. Three readings were taken at 15-min intervals with participants seated comfortably, and the left arm resting on a flat surface. The average of these readings was used to determine systolic blood pressure (SBP) and diastolic blood pressure (DBP) blood pressure levels.

Participants were classified as hypertensive and reported if the average SBP exceeded 140 mmHg and/or the average DBP exceeded 90 mmHg. A participant was classified as hypertensive if they had a documented hypertension diagnosis from a registered medical professional or were taking prescribed antihypertensive medications at the time of data collection. Additionally, individuals with average SBP > 140 mmHg and/or average DBP > 90 mmHg during data collection and not currently on antihypertensive medication were also considered hypertensive (Khan et al., 2021; Gabb et al., 2016).

### Outcome variables

The outcomes of the study were presence of anxiety and depression symptoms. The outcomes were evaluated using the Generalized Anxiety Disorder-7 (GAD-7) and Patient Health Questionnaire-9 (PHQ-9) scales, respectively. Both scales are popular worldwide in assessing the presence of anxiety and depression symptoms (Wu et al., 2025). Further, both scales had been previously validated among general population in Bangladesh (Redwan et al., 2020; Naher et al., 2021).

The GAD-7 scale comprises seven questions. Following the survey, the seven items of the GAD-7 scale demonstrated good internal consistency, with a Cronbach’s *α* of 0.74. Participants were asked to have anxiety symptoms in 2 weeks prior to the interview a 4-point Likert scale with options 0 (not at all), 1 (several days), 2 (more than half the days), and 3 (nearly every day). Considering the standard cut-off for Bangladeshi population, it was further coded binary as 0 for no or minimal anxiety symptoms (score: ≤10) and 1 coded for anxiety symptoms for rest of the categories (score: >10) (Redwan et al., 2020; Wahid et al., 2023).

Similarly, while assessing the presence of depression symptoms, the PHQ-9 scale consists of nine questions. Following the survey, the nine items of the GAD-7 scale showed acceptable internal consistency, with a Cronbach’s *α* of 0.69. Participants were asked to have depressive symptoms in 2 weeks prior to the interview using a 4-point Likert scale with options 0 (not at all), 1 (several days), 2 (more than half the days), and 3 (nearly every day). Adding up scores for individual question makes a total score of 27. Following the previous recommendation, the coding was extended to binary form: 0 denoted absence or minimal presence of depression symptoms (score: ≤10), while 1 indicated the presence of depression symptoms (score: >10) (Wahid et al., 2023; Manea et al., 2012).

The GAD-7 and PHQ-9 are widely used self-reported screening tools designed to identify individuals who may be experiencing symptoms of anxiety or depression symptoms. They serve as an initial step in determining the need for a more comprehensive clinical evaluation. In contrast, the diagnosis of anxiety and depression is typically conducted by clinicians, requiring specialized expertise in mental health.

### Independent variables

This study used demographic characteristics, including age, sex, educational status, employment status and marital status; behavioral factors, such as chewing tobacco, and smoking history; anthropometric characteristics such as, body mass index (BMI), and waist-hip ratio; clinical characteristics such as diabetes, CVD, other chronic diseases, and family history of hypertension. The majority of the independent variables have been identified as significant in previously published literatures (Nishat et al., 2023; Rois et al., 2021; Mridha et al., 2021; Islam et al., 2021; Mehareen et al., 2021; Hosen et al., 2021; Anjum et al., 2022). Detail operational definitions and scale of measurements of independent variables were presented in Supplementary Table S1.

### Statistical analysis

Baseline characteristics were assessed through descriptive analysis. The relationship between independent and dependent variables was examined using a Chi-square test, with significance considered when a *p*-value below 0.05 was achieved.

### ML model development and variable selection

All 13 selected independent variables were entered into the Extreme Gradient Booster (XGB) ML algorithm. The XGB algorithm, utilized in many studies, uses the power of ML techniques that selects variables after model fit (Chen and Guestrin, 2016; Zhao et al., 2023; Mortazavi et al., 2019).

The XGB algorithm is used over more interpretable traditional models despite the small dataset due to its ability to capture complex, nonlinear relationships and interactions among predictors that simpler models might miss (Xu et al., 2023). While traditional models like logistic regression are easier to interpret, they often assume linearity and independence among features, which may not adequately reflect real-world patterns in clinical or behavioral data. Ensemble methods, such as XGB, combine multiple weak learners to improve predictive performance, reduce overfitting, and enhance robustness, even in smaller datasets—especially when careful cross-validation and tuning are applied. In this context, improving prediction accuracy and identifying nuanced patterns took precedence over model simplicity to support more reliable decision-making (Ganie et al., 2025).

To develop both the XGB and LR model, a 5-fold nested cross-validation approach was implemented. In this approach, the dataset was randomly split into five subsets, and the process was repeated five times, with each subset serving as the test set once while the remaining four were used for training. The models’ performance was averaged across all iterations and evaluated on the unseen 20% test data. Thereafter, the results of the XGB model were compared to those of the LR model. To handle the class imbalance of outcome Adaptive Synthetic (ADASYN) resampling technique was used.

**Figure 2:**
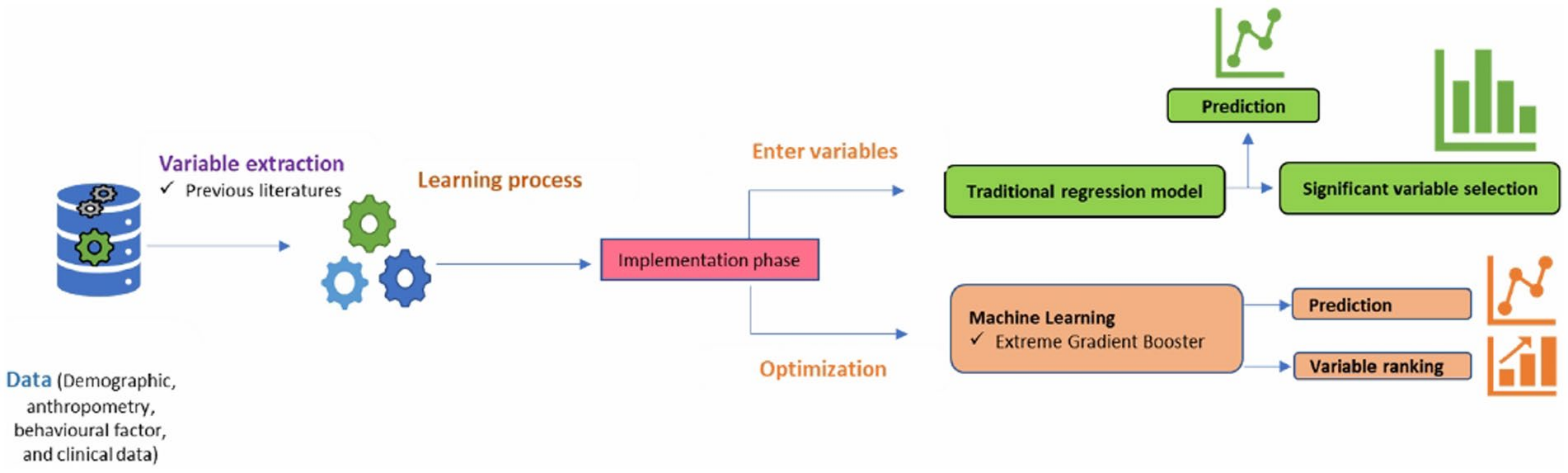
Machine learning model development.

The XGB model explained and ranked most influential factors using SHapley Additive exPlanations (SHAP) technique which was developed by Lundberg and Lee, incorporated principles from game theory and local explanation methodologies to evaluate the influence of individual features on a model’s decision-making process (Scott and Su-In, 2017). Further, odds ratio (OR) with 95% confidence interval (CI) and *p*-value were estimated for selected factors using traditional LR model. Detailed description of the model development was found in Figure 2.

### Model performance

The assessment and comparison of prediction performances of the XGB and LR models were determined by evaluating various metrics, including the accuracy, sensitivity, specificity, precision, recall, and F1 score (Islam et al., 2023; Monaghan et al., 2021). Models’ discrimination was assessed using the receiver operating characteristic (ROC) curve (Hajian-Tilaki, 2013). Model calibration was evaluated by examining the Brier score and calibration curve (Rufibach, 2010; Van Calster et al., 2019).

All data analysis was carried out using statistical software packages, specifically Stata (version 17), and Python 3.12.3.

## Results

### Background profile

Approximately 40.7% of rural hypertensive patients were aged 50 years or older, with 51% being male. Around 33.3% of respondents had no formal education, and 22.8% reported chewing tobacco. Additionally, 73.4% were classified as overweight or obese, 14.3% had diabetes, and 9.5% had some form of CVD. Furthermore, 36.9% of participants reported a family history of hypertension. A comprehensive background profile is detailed in Table 1.

**Table 1:** Background characteristics of the respondents.

Factors	Frequency	Percentages
*Demographic characteristics*		
Age (in years)		
30 and less	51	10.3
31–50	243	49.0
50 and above	202	40.7
Sex		
Male	243	49.0
Female	253	51.0
Educational status		
No formal education	165	33.3
Primary	125	25.2
Secondary	156	31.4
Higher	50	10.1
Employment status		
Employed or self-employed	195	39.3
Housewife	234	47.2
Retired or student	67	13.5
Marital status		
Never married, separated, divorced, or widowed	54	10.9
Currently married	552	89.1
*Behavioral factors*		
Chewing tobacco		
Never and past smoker	383	77.2
Current smoker	113	22.8
Smoking history		
Never and past user	431	86.9
Current user	65	13.1
*Anthropometric characteristics*		
BMI		
Normal and underweight	132	26.6
Overweight/pre-obesity	211	42.5
Obese	153	30.9
Waist-hip ratio (measurement)		
Low	90	18.2
Moderate	92	18.5
High	314	63.3
*Clinical characteristics*		
Diabetes		
No	425	85.7
Yes	71	14.3
Cardiovascular diseases		
No	449	90.5
Yes	47	9.5
Other chronic disease		
No	460	92.7
Yes	36	7.3
Family history of hypertension		
No	313	63.1
Yes	183	36.9

BMI, body mass index.

### Prevalence of anxiety and depression symptoms

The prevalence of symptoms of anxiety was 5.9% among rural individuals with hypertension, with 6.4% experienced depression symptoms. Anxiety symptoms were significantly more common among individuals with higher education (14.0%), those who were normal weight or underweight (9.9%), those with some form of CVDs (12.8%), and those with a family history of hypertension (8.7%). Similarly, symptoms of depression were significantly more prevalent among female hypertensive patients (8.7%), current tobacco users (12.4%), or individuals with CVDs (10.6%) and those with a family history of hypertension (9.8%) (Table 2).

**Table 2:** Prevalence of the presence of anxiety and depression symptoms.

Factors	Anxiety		Depression	
	*N* (%)	*p*-value	*N* (%)	*p*-value
Age (in years)				
30 and less	5 (9.8)	0.448	5 (5.9)	0.934
31–50	13 (5.4)		15 (6.2)	
50 and above	11 (5.4)		14 (6.9)	
Sex				
Male	12 (4.9)	0.392	10 (4.1)	0.038
Female	17 (6.8)		22 (8.7)	
Educational status				
No formal education	7 (4.2)	0.049	12 (7.3)	0.377
Primary	8 (6.4)		9 (7.2)	
Secondary	7 (4.5)		6 (3.9)	
Higher	7 (14.0)		5 (10.0)	
Employment status				
Employed or self-employed	9 (4.6)	0.606	9 (4.6)	0.199
Housewife	15 (6.4)		20 (8.6)	
Retired or student	5 (7.5)		5 (4.5)	
Marital status				
Never married, separated, divorced, or widowed	4 (7.4)	0.608	5 (9.3)	0.374
Currently married	25 (5.7)		27 (6.1)	
Smoking history				
Never and past smoker	28 (6.5)	0.112	29 (6.7)	0.518
Current smoker	2 (1.5)		3 (4.6)	
Chewing tobacco				
Never and past user	20 (5.2)	0.278	18 (4.7)	0.026
Current user	9 (8.0)		14 (12.4)	
Body mass index				
Normal and underweight	13 (9.9)	0.035	11 (8.3)	0.432
Overweight/pre-obesity	12 (5.7)		14 (6.6)	
Obese	5 (2.6)		7 (4.6)	
Waist-hip ratio (measurement)				
Low	5 (5.6)	0.954	3 (2.2)	0.195
Moderate	6 (6.5)		7 (7.6)	
High	18 (5.8)		23 (7.3)	
Diabetes				
No	24 (5.6)	0.621	27 (6.3)	0.827
Yes	5 (7.1)		5 (7.0)	
Cardiovascular diseases				
No	23 (5.1)	0.034	27 (6.0)	0.042
Yes	6 (12.8)		5 (10.6)	
Other chronic disease				
No	27 (5.9)	0.936	31 (6.7)	0.351
Yes	5 (5.6)		2 (2.8)	
Family history of hypertension				
No	13 (4.2)	0.036	14 (4.5)	0.019
Yes	16 (8.7)		18 (9.8)	
Total	29 (5.9)		32 (6.4)	

BMI, body mass index.

### Selection of variables and models’ performance comparison

**Figure 3:**
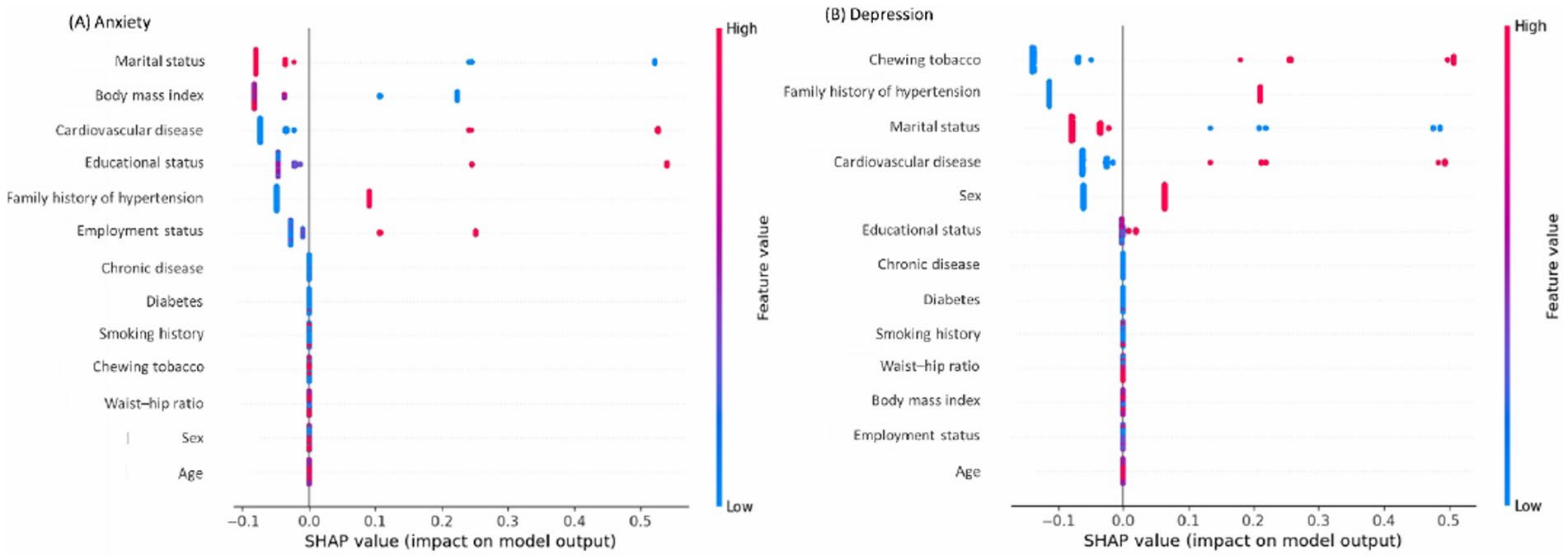
Most influential variables associated with **(A)** anxiety symptoms and **(B)** depression symptoms.

The XGB model identified marital status, BMI, CVD, educational status, family history of hypertension, and employment status as the most influential factors in predicting the presence of anxiety symptoms (Figure 3A). In Figures 3A,B, the vertical bar on the right illustrates the range of independent variable values, transitioning from lower (blue) to higher (red). SHAP values reflect the influence of independent variables on the model’s outcome. Variables with positive SHAP values on the *x*-axis indicate a positive association with the outcome, while those with negative SHAP values suggest a negative association. For instance, the variable marital status is binary, coded as 0 for individuals who are never married, separated, divorced, or widowed, and 1 for those currently married. In Figure 3A, blue represents a value of 0, while red represents a value of 1. Examining marital status in Figure 3A reveals that individuals who were not currently married (i.e., never married, separated, divorced, or widowed) (OR:1.01, 95% CI: 0.31–3.33) (Supplementary Table S2) were more likely to exhibit symptoms of anxiety, as indicated by the blue dots crossing 0 onwards on the SHAP value axis.

Similarly, being normal or underweighted people with hypertension (OR: 4.70, 95% CI: 1.44–15.34), hypertensive patients with CVD (OR: 3.33, 95% CI: 1.19–9.32), those who had higher education (OR: 4.22, 95% CI: 1.24–14.35), had family history of hypertension (OR: 2.25, 95% CI: 1.01–5.02), and currently not in employment status (OR:1.24, 95% CI: 0.36–4.32) had higher chance of getting the presence of anxiety symptoms (Figure 3 and Supplementary Table S2).

Concurrently, for the presence of depression symptoms, the XGB model revealed that current use of chewing tobacco (OR: 4.02 95% CI: 1.76–9.19), had family history of hypertension (OR: 2.12, 95% CI: 0.99–4.51), currently not in marital status (OR: 1.19, 95% CI: 0.41–3.45), had CVD (OR: 2.09, 95% CI: 0.71–6.14), female sex (OR: 3.10, 95% CI: 1.29–7.42), and those who had higher education had higher chance of getting the condition (OR: 3.22 95% CI: 0.91–11.38) (Figure 3B and Supplementary Table S3).

**Figure 4:**
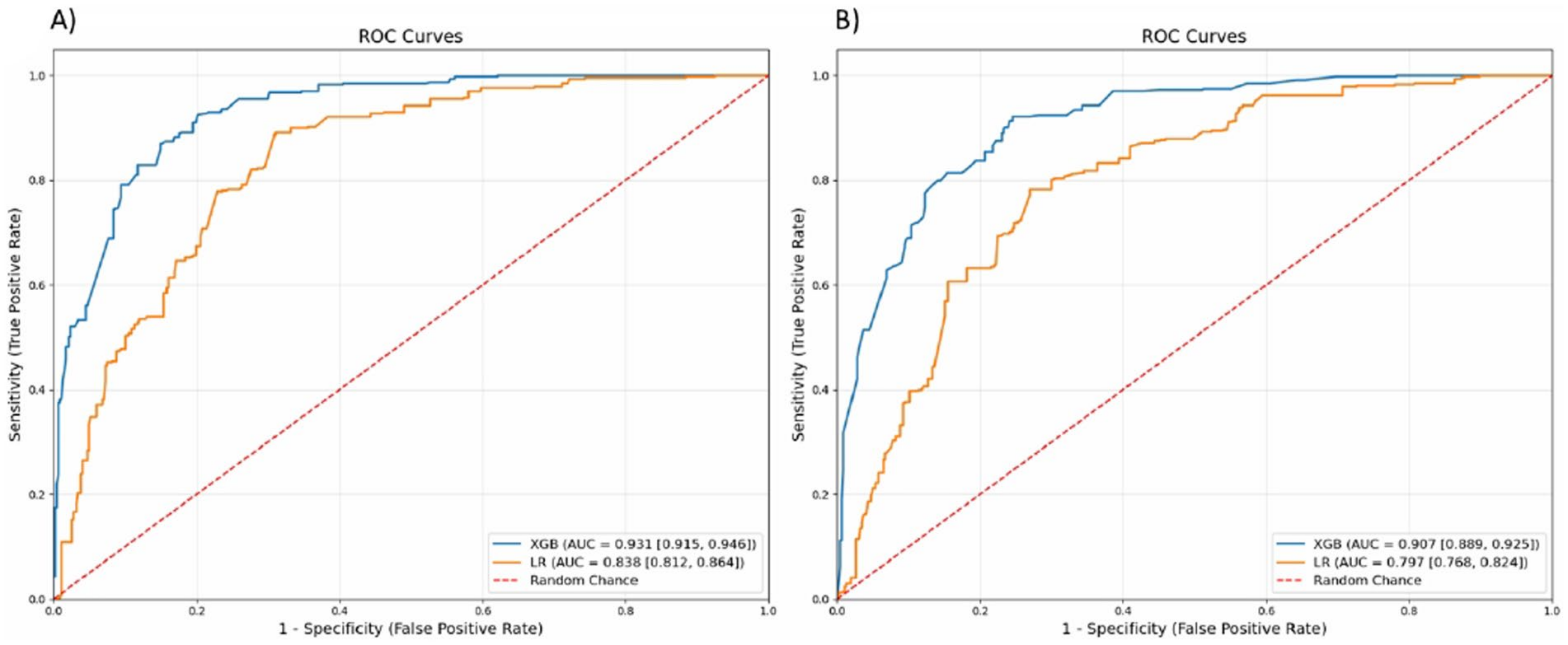
Receiver operating characteristics (ROC) curves as discrimination metrics for **(A)** anxiety symptoms and **(B)** depression symptoms. LR, logistic regression; XGB, extreme gradient booster.

The XGB model demonstrated improved performance over the traditional LR model in all metrics for predicting the presence of anxiety symptoms (Table 3). The XGB model achieved a high accuracy of 85.6% compared to 76.9% for the LR model. Notably, various performance metrics favored the XGB model, including sensitivity (95.5% for XGB vs. 90.0% for LR), specificity (71.7% for XGB vs. 64.7% for LR), precision (86.0% for XGB vs. 79.0% for LR), F1 score (83.0% for XGB vs. 77.0% for LR) and Brier score (14.4% for XGB vs. 23.1% for LR) (Table 3). The ROC score for predicting the presence of anxiety symptoms was notably higher for the XGB model at 93.1% (95% confidence interval (CI): 91.5–94.6%) compared to the LR model at 83.8% (95% CI: 81.2–86.4%) (Table 3 and Figure 4A).

**Table 3:** Models’ performance in predicting anxiety and depression symptoms.

Models’ performance	Models for symptoms of anxiety		Models for symptoms of depression	
	XGB	LR	XGB	LR
Models’ metrics				
Accuracy	0.856	0.769	0.834	0.750
Sensitivity	0.955	0.900	0.924	0.875
Specificity	0.717	0.647	0.692	0.556
Precision	0.860	0.790	0.830	0.740
F1 score	0.830	0.770	0.810	0.710
Calibration				
Brier score	0.144	0.231	0.166	0.250

CI, confidence interval, LR, logistic regression; ROC, receiver operating characteristics, XGB, extreme gradient booster. Higher the values of models’ metrics and ROC, better the prediction. F1 score is a measure of a model’s accuracy, which balances both precision and recall. High F1 score indicates both high precision and high recall. A low F1 score suggests either low precision, low recall, or both. A lower Brier Score indicates better prediction accuracy. It measures the mean squared difference between the predicted probabilities and the actual outcomes.

The XGB model outperformed the traditional LR model in all metrics for predicting the presence of depression symptoms (Table 3). The accuracy of the XGB model was 83.4% compared to 75.0% for the LR model. Notably, other performance metrics were higher for the XGB model, including sensitivity (92.4% for XGB vs. 87.5% for LR), specificity (69.2% for XGB vs. 55.6% for LR), precision (83.0% for XGB vs. 74.0% for LR), F1 score (81.0% for XGB vs. 71.0% for LR) and Brier score (16.6% for XGB vs. 25.0% for LR) (Table 3). The ROC score for predicting the presence of depression symptoms was higher for the XGB model at 90.7% (95% CI: 88.9–92.5%) compared to the LR model at 79.7% (95% CI: 76.8–82.4%) (Table 3 and Figure 4B).

**Figure 5:**
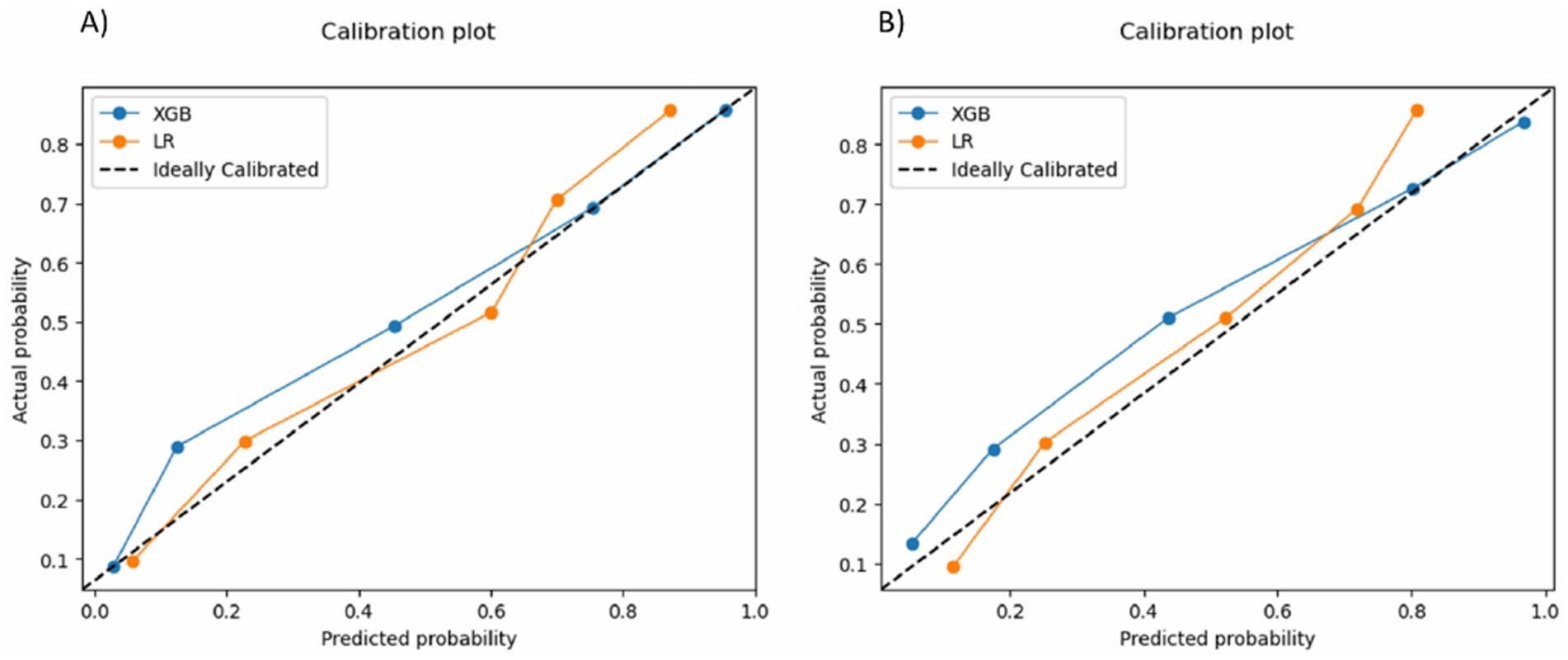
Calibration curves for **(A)** anxiety symptoms and **(B)** depression symptoms. LR, logistic regression; XGB, extreme gradient booster.

For both outcomes, the calibration curve of the XGB model closely followed the ideal line for the presence of anxiety and depression symptoms, demonstrating near-perfect calibration (Figure 5).

## Discussion

In rural areas of Bangladesh, the prevalence of anxiety and depression symptoms among people with hypertension was 5.9% and 6.4%, respectively, which was similar to the national prevalence among the general population (NIMH, 2019). However, the prevalence was notably higher among this subgroup in some neighboring countries such as Afghanistan and Nepal (Hamrah et al., 2018; Shah et al., 2022). Given the current status of these psychiatric conditions, this study investigated the factors associated with anxiety and depression symptoms in this population using a sophisticated ML method and compared the results with the traditional LR model. In line with this, the study showed that the ML-based XGB model outperforms traditional LR model in determining the presence of anxiety and depression symptoms, while also revealing a notable overlap in identifying associated factors.

In this study, the XGB model demonstrated improved performance over the traditional LR model in predicting the presence of anxiety and depression symptoms across all performance metrics. For both outcomes, the XGB model showed higher accuracy, sensitivity, specificity, precision, F1 score, brier score and ROC score than the LR model. These findings align with prior research, highlighting the potential of ML models to improve the prediction of anxiety and depression symptoms in clinical settings (Chekroud et al., 2016; Kessler et al., 2016).

After demonstrating the potential of the ML approach, the current study also investigated various factors associated with the presence of anxiety and depression symptoms among rural individuals with hypertension, as identified by the XGB model. In predicting the presence of anxiety symptoms, the XGB model identified marital status, BMI, CVD, educational status, family history of hypertension and employment status as influential contributors for individuals with hypertension. The findings partially align with previous studies conducted in Afghanistan and Ethiopia, where marital status, occupation, and chronic comorbid illnesses were identified as significant factors associated with the presence of anxiety symptoms among individuals with hypertension (Abdisa et al., 2022; Hamrah et al., 2018). However, unlike the present study, those previous studies identified sex and smoking as significant contributors to anxiety in their study subgroups. Additionally, prior studies in Bangladesh among diverse subgroups such as school adolescents, married female adolescents, rural pregnant women, and university students also highlighted the importance of factors such as age, education, marital status, smoking, alcohol use, chronic illness, and employment status as influential factors for anxiety (Nishat et al., 2023; Nasreen et al., 2011; Islam et al., 2021; Anjum et al., 2022).

Regarding the presence of depression symptoms, the XGB model identified chewing tobacco, family history of hypertension, marital status, CVD, sex, and educational status as the top contributing factors. These findings partially align with previous studies conducted in Afghanistan, Ethiopia, and Morocco, which identified sex, education, marital status, tobacco use, chronic comorbidities, and family history of hypertension as significant factors associated with depression symptoms among individuals with hypertension (Abdisa et al., 2022; Hamrah et al., 2018; Boukhari et al., 2024).

In contrast, previous studies in Bangladesh consistently found factors such as sex, education level, BMI, smoking, tobacco use, and self-reported health conditions to significantly contribute to depression across various population subgroups, including adolescents, university students, healthcare workers, and type 2 diabetes patients, regardless of the use of ML (Mridha et al., 2021; Mehareen et al., 2021; Hossen et al., 2024; Haque et al., 2023). Additionally, an ML-based study identified smoking as a key factor influencing depression among university students in Bangladesh which was inconsistent with the present study (Rois et al., 2021).

However, previous ML-based studies on predicting anxiety and depression conditions across different population subgroups were largely focused on methodology and did not extensively investigate associated factors (Moon et al., 2021; Nayan et al., 2022; Choudhury et al., 2019). Furthermore, the lack of studies in this specific subgroup presents an opportunity to gain deeper insights into this issue. The application of ML in predicting associated factors associated with psychiatric symptoms for anxiety and depression has the potential to significantly contribute to the planning of strategies and formulation of policies or revisiting the existing strategies to reduce the burden of psychiatric conditions, particularly among rural hypertensive patients.

This study also found that educational status, marital status, CVD and family history of hypertension were common factors associated with both anxiety and depression symptoms among rural individuals with hypertension, as identified by the XGB model. Previous studies have supported these findings, regardless of the hypertensive cohort studied (Islam et al., 2021; Hosen et al., 2021; Hossen et al., 2024; Haque et al., 2022). Among these common factors, having a higher level of education while living with hypertension can contribute to anxiety and depression due to a complex interplay of various influences. Physical health challenges, such as the stress of constantly monitoring blood pressure, can heighten anxiety, while psychological factors, including feelings of stigma in professional or academic settings, may exacerbate mental health struggles. Societal expectations, such as the pressure to succeed in both personal and professional life, can further add to this burden. Additionally, individuals with hypertension who are unmarried, separated, divorced, widowed, or widowers may experience social isolation or societal stigma, further increasing their risk of anxiety and depression (Subramaniam et al., 2017). Moreover, managing a chronic disease, whether as an affected individual or a family member, can create significant emotional strain, potentially leading to mental health issues (Huang et al., 2023; Gallo et al., 2021).

Based on the findings and their potential consequences, this research suggests several strategies, including promoting mental health education and counseling, and developing mechanisms to facilitate mental health support in rural areas. These include stress management education and coping strategies to address psychological barriers; designing a counseling program that provides a safe space for patients to express their feelings, fears, and concerns, thereby reducing anxiety and improving overall wellbeing; empowering patients by clarifying the relationship between hypertension and mental health risks, enabling them to take control of their health and make informed decisions; and facilitating counseling to encourage behavioral changes, such as adopting a healthier diet, engaging in regular exercise, and quitting smoking or tobacco use. Integrating mental health education and counseling into hypertension management can lead to better health outcomes, improved quality of life, and a more holistic approach to healthcare.

Besides making a notable impact on the rural community, the main strength of this study is its successful implementation of a robust ML model. Moreover, it provides a comprehensive evaluation of factors associated with psychiatric symptoms among rural hypertensive patients, utilizing a systematically collected sample. However, a notable limitation is that the data were gathered retrospectively and relied on self-reporting, which introduces the potential for underreporting and recall bias. Variables collected for different purposes may limit the inclusion of important behavioral factors such as sleep problems, mobile phone use, physical activity, eating behaviors, the use of anti-hypertensive drugs, and the control or stability of blood pressure. Additionally, the cross-sectional nature of the study prevents the establishment of causal associations. The small sample size may not be representative, potentially affecting generalizability. Furthermore, the ML models employed lack the capability to generate significance level or *p*-values for examining the associations between independent and dependent variables.

## Conclusion

In rural Bangladesh, around 6% hypertensive patients exhibited symptoms of anxiety and depression. The application of the XGB model in determining the presence of anxiety and depression symptoms outperformed the traditional LR model. Educational status, marital status, CVD, and family history of hypertension were the top factors associated with both anxiety and depression symptoms among individuals with hypertension. Future research in this field may benefit from employing ML models, which could enhance early detection, support mechanisms, informed decision-making, and policy formulation.

## Data Availability

The original contributions presented in the study are included in the article/Supplementary material, further inquiries can be directed to the corresponding author.
