# Supplementary material for: Factors associated with the presence of anxiety and depression symptoms in rural hypertensive adults in Bangladesh: leveraging extreme gradient booster machine learning algorithm
Source: Front Psychol. 2025 Sep 10;16:1650667. doi: 10.3389/fpsyg.2025.1650667 (PMC12457397; doi:10.3389/fpsyg.2025.1650667)
Supplement: Supplementary file 1 [file Table_1.docx]

**Supplementary Text S1. Kish Grid method**

It is a method used in survey research to obtain representative samples from a population across a geographic area. This technique involves creating a list of eligible individuals at a specific address, arranged by age, and then selecting participants based on the address's serial number. The system is designed to ensure that everyone in a household has an equal chance of being selected. By doing so, this method guarantees randomness, minimizes bias, and covers the entire population, making it an efficient approach for sampling geographically dispersed populations during survey research and data collection. However, researchers must consider factors such as grid size, resolution, and potential clustering effects to implement this method effectively.

**Supplementary Text S2. Recruitment and training workshop for field personnel**

We have appointed five interviewers (along with two local support persons) with a minimum graduate-level education with social science backgrounds and previous experienced in data collection as ad hoc basis. A registered medical nurse was appointed and trained to measure anthropometrics and blood pressure. The chief investigator primary organized a six-day one week online (Zoom) workshop for the interviewers to familiarise them with this study’s objective and interview questionnaire. Additionally, two support persons have been recruited from the local area of the data collection site. They helped the team navigate the area and build rapport with local residents. Mock interviews were conducted as a pre-test to ensure the interviewers were able to practice relevant techniques and adapt to difficult situations during the main interview.

**Supplementary Text S3. Data collection instrument**

Data was collected by face-to-face interviews using a validated semi-structured questionnaire. The original questionnaire was in English, which was translated into Bengali for the convenience of local comprehension facilitating data collection. The Bengali version was then converted in to English to ensure linguistic and conceptual equivalency. The survey questionnaire had the following sections: participants’ demographic and life-style factors; and participants’ clinical data, which includes hypertension related questions. As a component of face validity, the research team subjectively evaluated the questionnaires presentation and relevance to ensure the items were pertinent, reasonable, unambiguous, and clear. Additionally, content validity was ensured through a comprehensive literature review, followed by evaluation from an expert panel in the relevant research field.

**Supplementary Text S4. Pilot study**

A pilot study was conducted on 24 participants from the selected sampling area to check the acceptability and feasibility of the questionnaire and the average time required to complete the survey. After the pilot study, no major modification was made to the questionnaire except a few minor languages change. The data from pilot study was not included in the study sample. The questionnaire took approximately 30 min to complete per participant.

**Supplementary Text S5. Quality assurance**

To ensure that the quality of data collection was maintained, we have applied several measures: (i) prior the survey, the questionnaire underwent pre-testing to identify and rectify inconsistencies, unclear language, or prolonged administration times. Following appropriate modifications, the questionnaire was finalised; (ii) pre-survey workshop organised for field personnel to outline the purpose of the study, the procedures and potential difficulties related with data collection; (iii) maintained strict monitoring of the data collection and management processes were regularly monitored by one of the local senior investigators; and (iv) utilisation of resilient equipment for physical and clinical measurements. The investigators carried out a random consistency check for at least 5% of the interviewed questionnaires.

**Supplementary Text S6. Safety measure**

Data collection took place during the COVID-19 pandemic, following strict safety protocols. All data collectors and support staff wore face masks, maintained a 1.5-meter distance, and used hand sanitizer before and after each procedure. Interviewees were also provided with face masks and encouraged to use hand sanitizer. Data collection began only after the Government of Bangladesh relaxed restrictions.

**Supplementary Text S7.** **Data access and storage**

Google spreadsheet was used for data entry, editing, sorting and coding of the collected data. The final spreadsheet has shifted to Stata software and saved in the secure faculty-allocated network storage (Monash (S:) drive) as a backup. Only the research team (chief investigator and co-investigators) will have access to these electronic databases. The data were stored in both REDCap and Monash (S:) drive for five years as per Monash University policy after which it will be permanently deleted.

**Supplementary Table S1** Measurements of independent variables

| **Variable** | **Description** | **Measurement** | **Scale of measurement** |
| --- | --- | --- | --- |
| **Demographic variables** | | | |
| Age | Participants age in years during the data collection period | <35, 35-50 and ≥51 | Categorial |
| Sex | Sex of the participants | Male, female | Binary |
| Educational status | Educational status can be categorised as "educated" when they have completed a minimum of five years of schooling, typically corresponding to the primary (class 1 to 5), secondary (class 6 to 10), and/or higher level. On the other hand, if there are no schooling years completed (0 years of schooling), this categorised them as "no formal education." | No formal education, primary, secondary, or higher | Categorical |
| Employment status | Participants engaged in economic activity at the time of data collection. | Employed or self-employed, housewife, retired or student (currently not in employment status) | Categorical |
| Marital status | Participant’s marital relationship status during the data collection period. | Never married, separated, divorced, or widowed, and currently married | Categorical |
| **Lifestyle factors** | | | |
| Chewing tobacco | Adaptable behaviours and ways of life like influence the participant’s health and well-being. | Past and non-user, current user | Binary |
| Smoking history | Adaptable behaviours and ways of life like influence the participant’s health and well-being. | Past and non-smoker, current smoker | Binary |
| **Anthropometric data** | | | |
| Body mass index (BMI) | BMI was derived from the measured values of height and weight. Using WHO’s BMI guidelines for the Asian population, BMI has been classified. **Height** was measured using a portable stadiometer (Seca 213 Portable Stadiometer) with light clothing and while barefoot. Study participants were instructed to stand straight against a wall looking forward with loosely hanging arms on their sides. The head plate was put on the crown of the head and a measurement on tape was noted to the nearest 0.5 cm. The measurement was taken twice. If the difference was more than 2 mm, a third measurement was taken. The average of the measurements was recorded as the participant's height.  A third measurement was taken if the difference was more than 2 mm. The average of the measurements was recorded as the participant’s height. Further, participants’ **weight** was measured using a digital weighing scale (BEURER wellbeing PS 240*)* with light clothing and while barefoot. Participants were instructed to stand still in the center of the machine looking forward with loosely hanging arms on their sides. Weight was recorded to the nearest 0.1 kg. | Underweight (<18.50 kg/m^2^), normal (18.50-22.99 kg/m^2^), overweight (23.00-27.49 kg/m^2^), and obese (≥27.50 kg/m^2^) | Categorical |
| Waist–hip ratio | The waist–hip ratio is the dimensionless ratio of the circumference of the waist to that of the hips. This was calculated as waist circumference (WC) divided by hip circumference (HC) (WC⁄HC). Optimal cut-off values of waist–hip ratio was measured. The cut-off points are 85 cm for Asian men and 75–80 cm for Asian women and categorised as low, moderate and high. Participants’ WC was measured while they were wearing thin clothing (if they were clothed for cultural reasons), on exhalation, midway between the lower rib margin and the anterior superior iliac spine (hip bone), or the narrowest abdominal point. Participants were relaxed with arms hanging loosely by their sides. The tape measure was kept horizontal for a standing measurement. This was done twice. If the measurements differed by more than 2 cm, a third measurement has been taken. The average of the measurements was recorded as the participant’s WC. Further, a measurement was taken at the widest circumference around the hip bones so that the tape passes over the greatest protrusion of the gluteal muscles. The tape measure was kept horizontal for a standing measurement. This was done twice. If the measurements differed by more than 2 cm, a third measurement was taken. The hip circumference was recorded to the nearest 0.5 cm. | Low, moderate and high | Categorical |
| **Clinical characteristics** | | | |
| Diabetes | Participant living with diabetes mellitus was considered if there was a documented diagnosis and/or medication history. | No, yes | Binary |
| Cardiovascular diseases | Patients had heart attack and stroke received treatment and/or were taking prescribed medication. | No, yes | Binary |
| Other chronic disease | Chronic diseases such as kidney disease, asthma, cancer, arthritis or others were considered to be present if there was a documented diagnosis, medication history, or any past procedures. | No, yes | Binary |
| Family history of hypertension | Any blooded family member had high blood pressure | No, Yes | Binary |

**Supplementary** **Table S2**. Association between anxiety symptoms and selected factors by XGB model

| **Variables** | OR (95% CI) | p-value |
| --- | --- | --- |
| **Marital status** |  |  |
| Never married, separated, divorced or widowed | 1.01 (0.31 - 3.33) | 0.984 |
| Currently married | 1.00 |  |
| **Body mass index** |  |  |
| Normal and underweight | 4.70 (1.44 - 15.34) | 0.010 |
| Overweight/pre-obesity | 2.52 (0.77 - 8.24) | 0.126 |
| Obese | 1.00 |  |
| **Cardiovascular diseases** |  |  |
| No | 1.00 |  |
| Yes | 3.33 (1.19 - 9.32) | 0.022 |
| **Educational status** |  |  |
| No formal education | 1.00 |  |
| Primary | 1.35 (0.45 - 4.01) | 0.594 |
| Secondary | 1.09 (0.36 - 3.35) | 0.880 |
| Higher | 4.22 (1.24 - 14.35) | 0.021 |
| **Family history of hypertension** |  |  |
| No | 1.00 |  |
| Yes | 2.25 (1.01 - 5.02) | 0.048 |
| **Employment status** |  |  |
| Employed or self-employed | 1.00 |  |
| Housewife | 1.76 (0.71 - 4.38) | 0.222 |
| Retired or student | 1.24 (0.36 - 4.32) | 0.731 |

**Supplementary** **Table S3**. Association between depression symptoms and selected factors by XGB model

| **Variables** | OR (95% CI) | p-value |
| --- | --- | --- |
| **Chewing tobacco** |  |  |
| Never and past smoker | 1.00 |  |
| Current smoker | 4.02 (1.76 - 9.19) | 0.001 |
| **Family history of hypertension** |  |  |
| No | 1.00 |  |
| Yes | 2.12 (0.99 - 4.51) | 0.052 |
| **Marital status** |  |  |
| Never married, separated, divorced or widowed | 1.19 (0.41 - 3.45) | 0.749 |
| Currently married | 1.00 |  |
| **Cardiovascular diseases** |  |  |
| No | 1.00 |  |
| Yes | 2.09 (0.71 - 6.14) | 0.178 |
| **Sex** |  |  |
| Male | 1.00 |  |
| Female | 3.10 (1.29 - 7.42) | 0.011 |
| **Employment status** |  |  |
| No formal education | 1.00 |  |
| Primary | 1.40 (0.52 - 3.81) | 0.505 |
| Secondary | 0.79 (0.27 - 2.33) | 0.665 |
| Higher | 3.22 (0.91 - 11.38) | 0.070 |
